# Antimicrobial susceptibility of *Legionella gormanii*: species-specific ECOFFs, distinct MICs for rifampin versus *Legionella pneumophila*

**DOI:** 10.1186/s12941-025-00813-8

**Published:** 2025-09-01

**Authors:** Jun-Wei Xu, Yuan-Tong He, Xiao-Yong Zhan

**Affiliations:** The Seventh Affiliated Hospital, Sun Yat-sen University, No.628, Zhenyuan Road, Guangming District, Shenzhen, 518107 China

**Keywords:** *Legionella gormanii*, *Legionella pneumophila*, Antimicrobial susceptibility, Macrolides, Fluoroquinolone, Tetracyclines, Rifampin, Trimethoprim-sulfamethoxazole

## Abstract

**Background:**

*Legionella gormanii (L. gormanii)* is an emerging pathogen causing legionellosis, yet it is much less studied than the predominant species, *L. pneumophila*. Clinical treatment guidelines for legionellosis are primarily based on data from *L. pneumophila* and recommend macrolides and fluoroquinolones. However, the intrinsic antimicrobial susceptibility of *L. gormanii* is not well-defined, creating uncertainty about whether these guidelines are directly applicable. Establishing a baseline understanding of its susceptibility patterns is a prerequisite for effective epidemiological surveillance and for identifying non-wild-type resistance. This study aims to address this fundamental knowledge gap by characterizing the in vitro susceptibility profiles of a large collection of environmental *L. gormanii* isolates.

**Methods:**

This study systematically evaluated the in vitro activity of ten representative antimicrobials against 207 environmental *L. gormanii* isolates collected in China between 2019 and 2021. Minimum inhibitory concentrations (MICs) were determined by the broth microdilution (BMD) method, and species-specific epidemiological cutoff values (ECOFFs) were established using the ECOFFinder tool.

**Results:**

Most tested agents demonstrated good in vitro activity. Rifampin was the most potent agent, while clarithromycin was the most active macrolide. Conversely, tetracyclines showed limited activity. Comparative analysis revealed that *L. gormanii* exhibited markedly higher MICs for rifampin (approximately 9.58-fold) than typically reported for *L. pneumophila*. Species-specific ECOFFs were determined for nine antimicrobials: rifampin (0.016 mg/L); ciprofloxacin, levofloxacin, and clarithromycin (0.063 mg/L); moxifloxacin (0.125 mg/L); erythromycin (0.25 mg/L); azithromycin (0.5 mg/L); trimethoprim-sulfamethoxazole (4 mg/L); and tigecycline (16 mg/L).

**Conclusions:**

This study establishes the first large-scale susceptibility dataset and species-specific ECOFFs for *L. gormanii*. The findings highlight key inter-species differences in susceptibility, particularly for rifampin, underscoring that treatment paradigms cannot be safely extrapolated from *L. pneumophila*. These ECOFFs provide a critical tool for surveillance of non-wild-type resistance. However, these data, derived from environmental isolates, are intended for epidemiological and hypothesis-generating purposes and must be supplemented with clinical and pharmacokinetic/pharmacodynamic (PK/PD) studies before informing changes to clinical practice.

**Supplementary Information:**

The online version contains supplementary material available at 10.1186/s12941-025-00813-8.

## Introduction

*L. gormanii*, a Gram-negative facultative intracellular pathogen, is an increasingly recognized etiological agent of atypical pneumonia [1]. While *Legionella pneumophila* is responsible for the majority of legionellosis cases globally, non-*pneumophila* species, including *L. gormanii*, are responsible for sporadic but sometimes severe cases of pneumonia, particularly in vulnerable populations [2–7]. The true epidemiological prevalence of *L. gormanii* is likely underestimated due to methodological constraints: standard diagnostic assays, such as urinary antigen tests, exhibit high specificity for *L. pneumophila* serogroup 1 but fail to detect most non-*pneumophila* species, leading to underdiagnosis and biased surveillance data [8, 9]. Despite its growing public health relevance, *L. gormanii* remains less understood than *L. pneumophila*, particularly regarding its antimicrobial susceptibility profiles. This knowledge gap undermines evidence-based therapeutic strategies, posing risks for suboptimal treatment outcomes.

Current legionellosis guidelines, largely extrapolated from *L. pneumophila* data, prioritize macrolides (e.g., azithromycin) and fluoroquinolones (e.g., levofloxacin) due to their intracellular penetration and bactericidal activity [10]. Nevertheless, interspecies variability in antimicrobial susceptibility is well-documented can arise from differences intrinsic resistance mechanisms including efflux pumps, target site mutations, or biofilm formation. Furthermore, the absence of a large collection of tested *L. gormanii* strains has thus far precluded the establishment of standardized ECOFFs, which are indispensable tools for distinguishing wild-type (WT) isolates from those that may have acquired resistance mechanisms, thereby facilitating resistance surveillance [11].

A preliminary epidemiological investigation of *Legionella* in the Chinese environment revealed a substantial prevalence of *L. gormanii* [12]. It accounted for 10.43% of total *Legionella* isolates, positioning it as the second most frequently isolated species after *L. pneumophila* (73.34%) [12]. The identification of severe pneumonia cases attributed to *L. gormanii* in China [2, 3], alongside its environmental abundance, underscores the potential public health risk associated with this species.

To address these gaps, this study presents a comprehensive analysis of the in vitro activity of ten key antimicrobial agents, spanning macrolides (erythromycin, azithromycin, clarithromycin), fluoroquinolones (levofloxacin, ciprofloxacin, moxifloxacin), tetracyclines (tigecycline, doxycycline), rifampin, and trimethoprim-sulfamethoxazole (TMP/SMX)—against 207 environmental *L. gormanii* isolates collected between 2019 and 2021 in China. Employing BMD methodology, we established species-specific MIC distributions and derived ECOFF values using non-parametric statistical modeling. Our findings provide a crucial foundation for antimicrobial stewardship, targeted surveillance, and future research into this emerging pathogen.

## Methods and materials

### *L. gormanii* isolates

A total of 207 *L. gormanii* isolates, procured through systematic environmental surveillance between April 2019 and October 2021 across ten geographically diverse municipalities in China were included in this study. These isolates originated from various aquatic (203 isolates) and soil environments (4 isolates), including lake water, river water, pond water, and soil samples. A detailed breakdown of isolate information is provided in Table S1. Isolates were cryopreserved at − 80 °C in buffered yeast extract (BYE) broth with 40% (v/v) glycerol.

### Antimicrobial agents

Antimicrobial susceptibility was evaluated using a panel of ten agents from five pharmacological classes. Fluoroquinolones (ciprofloxacin, CIP; moxifloxacin, MOX; and levofloxacin, LEV) were tested at concentrations ranging from 0.004 to 8 mg/L. Macrolides (erythromycin, ERY; clarithromycin, CLA; and azithromycin, AZI) were assessed within the same range as macrolides. TMP/SMX was tested at a fixed 1:5 mass ratio (ranging from 0.004 to 8 mg/L). Tetracyclines (doxycycline, DOX; and tigecycline, TIG) were evaluated at 0.016 to 32 mg/L, while rifampin (RIF) was tested separately at concentrations from 0.0000625 to 0.125 mg/L. All compounds were s procured from certified commercial suppliers: Shanghai Rhawn Chemical Technology Co., Ltd. (Shanghai, China) provided the fluoroquinolones, macrolides, and RIF, while Shanghai Macklin Biochemical Co., Ltd. (Shanghai, China) supplied the tetracyclines and TMP/SMX.

### Antimicrobial susceptibility testing

Antimicrobial susceptibility testing (AST) was performed using the BMD method. While agar-based methods, such as gradient strip or diffusion assays, can yield biased and inconsistent MICs for *Legionella* due to the adsorption of antimicrobial agents by charcoal [13, 14], BMD offers enhanced stability and provides unbiased MICs, and is considered a reliable and internationally recognized reference method for this genus [14–16]. Prior to AST, *L. gormanii* isolates were cultured on buffered charcoal yeast extract agar (BCYEα) supplemented with α-ketoglutarate (1 g/L), L-cysteine (0.4 g/L), and ferric pyrophosphate (0.25 g/L) under microaerophilic conditions (37 °C, 5% v/v CO₂, high humidity) for 72 h before AST. The AST protocol was adapted from established guidelines [17–19]. Briefly, a single colony was suspended in BYE broth and adjusted to an optical density (OD₆₀₀) of 0.1 (~ 1.0 × 10⁸ CFU/mL). Two-fold serial dilutions of antibiotics were prepared in 96-well microtiter plates, and the final well volume was 100 µL (containing 2 × 10⁴ CFU bacteria). This protocol, including the 100 µL volume, is consistent with high-throughput workflows and commercial systems, and potential evaporation was mitigated by incubation in a humidified atmosphere. Plates were incubated at 37 °C for 48 h. This incubation time was determined to be optimal in preliminary experiments, where extending incubation to 72–96 h did not alter the MIC results. The MIC was defined as the lowest antibiotic concentration that inhibited visible growth. Assays were performed in duplicate. *L. pneumophila* ATCC 33,152 was used as the quality control strain in all assay batches.

### Definition of MIC_50_, MIC_90_, and the wild-type *L. gormanii* ECOFF

The MIC_50_ and MIC_90_ values, representing the MICs inhibiting 50% and 90% of isolates, respectively, were determined to assess susceptibility distribution. The ECOFF was used to differentiate susceptible and resistant populations based on MIC distributions. ECOFFs were determined using the ECOFFinder program (version 2010-v21) from EUCAST [11, 20], which fits a log-normal distribution to MIC data. The 97.5th percentile of MIC values was designated as the ECOFF for each antibiotic against *L. gormanii*, following the principle that 97.5% of wild-type isolates fall within the defined range.

## Results

### MIC distribution patterns in *L. gormanii* isolates: quantitative ranges and cumulative Inhibition thresholds (MIC_50_/_90_)

The in vitro antimicrobial susceptibility profiles of 207 *L. gormanii* isolates against ten antibiotics were determined by MIC distributions (Table 1). Analysis revealed that most isolates were susceptible to low MICs of RIF, fluoroquinolones, and macrolides. RIF demonstrated the highest potency (geometric mean MIC = 0.0030 mg/L), while DOX showed the lowest susceptibility (geometric mean MIC = 6.244 mg/L). Fluoroquinolones exhibited comparable MIC distributions (geometric mean MICs: 0.0198 to 0.0315 mg/L). Tetracyclines (DOX and TIG) had overlapping MIC ranges (geometric mean MICs: 5.299–6.244 mg/L). Among macrolides, CLA was the most active (geometric mean MIC = 0.0125 mg/L), with approximately eight-fold lower MICs than AZI (geometric mean MIC = 0.0973 mg/L).

**Table 1:**
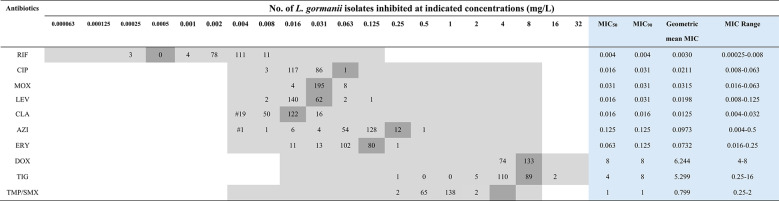
MIC_50_ and MIC_90_ values of tested antimicrobial agents and their susceptibility patterns in *L. gormanii*

The gray-shaded cells indicate the range of concentrations tested for each antimicrobial agent. The darker gray-shaded cells indicate the MIC values of the reference strain, L. pneumophila ATCC 33152, which was included as a quality control in each batch of AST and yielded consistent results across all experiments. MIC_50_, MIC_90_, geometric mean MIC, and MIC range are highlighted in light-blue cells

### Epidemiological cutoff values for *L. gormanii*

**Fig. 1:**
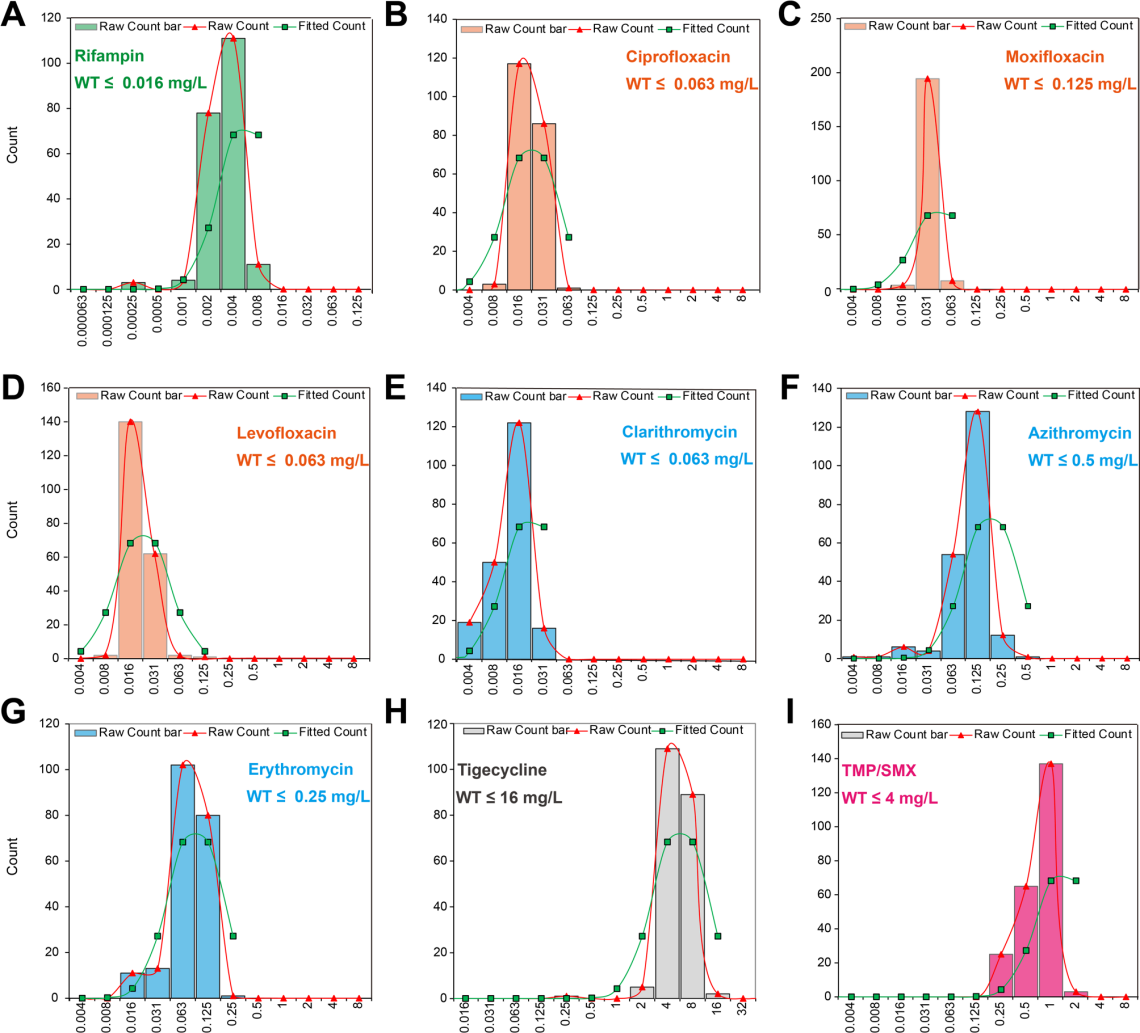
MIC Distributions and ECOFF Determination for nine antimicrobial agents against *L. gormanii*. ECOFFs, serving as the Wild-type Cutoffs (COWTs) for these agents in *L. gormanii* populations, were determined using the ECOFFinder method. Panels (**A**–**I**) display the corresponding MIC distribution for each of the nine tested antimicrobial agents. Each panel includes the raw count data histogram, the fitted curve generated by the ECOFFinder software, and the calculated ECOFF value (indicated as “WT ≤ [MIC] mg/L”)

Species-specific ECOFFs were determined for nine agents using the ECOFFinder software (Fig. 1). The analysis yielded the following ECOFFs: 0.016 mg/L for RIF, 0.063 mg/L for CIP, LEV, and CLA, 0.125 mg/L for MOX, 0.25 mg/L for ERY, 0.5 mg/L for AZI, 4 mg/L for TMP/SMX, and 16 mg/L for TIG. An ECOFF for DOX could not be established due to an insufficient MIC distribution (two data points) for valid statistical modeling. Crucially, the MICs for all 207 *L. gormanii* isolates were at or below the newly established ECOFFs for all tested agents. This provides strong evidence that acquired resistance mechanisms are currently absent in this large, diverse population of environmental isolates.

### Comparison of antimicrobial susceptibility profiles between *L. gormanii* and *L. pneumophila*

To benchmark the antimicrobial susceptibility of our *L. gormanii* isolates, a comparative analysis was conducted against published MIC data for *L. pneumophila*. To ensure methodological consistency, the comparative data were sourced exclusively from large-scale studies that employed the BMD method for MIC determination (Table 2). For each antimicrobial agent, up to four benchmark reports with the highest number of tested isolates were included, with their detailed MIC distributions provided in the supplementary materials (Table S2).

**Fig. 2:**
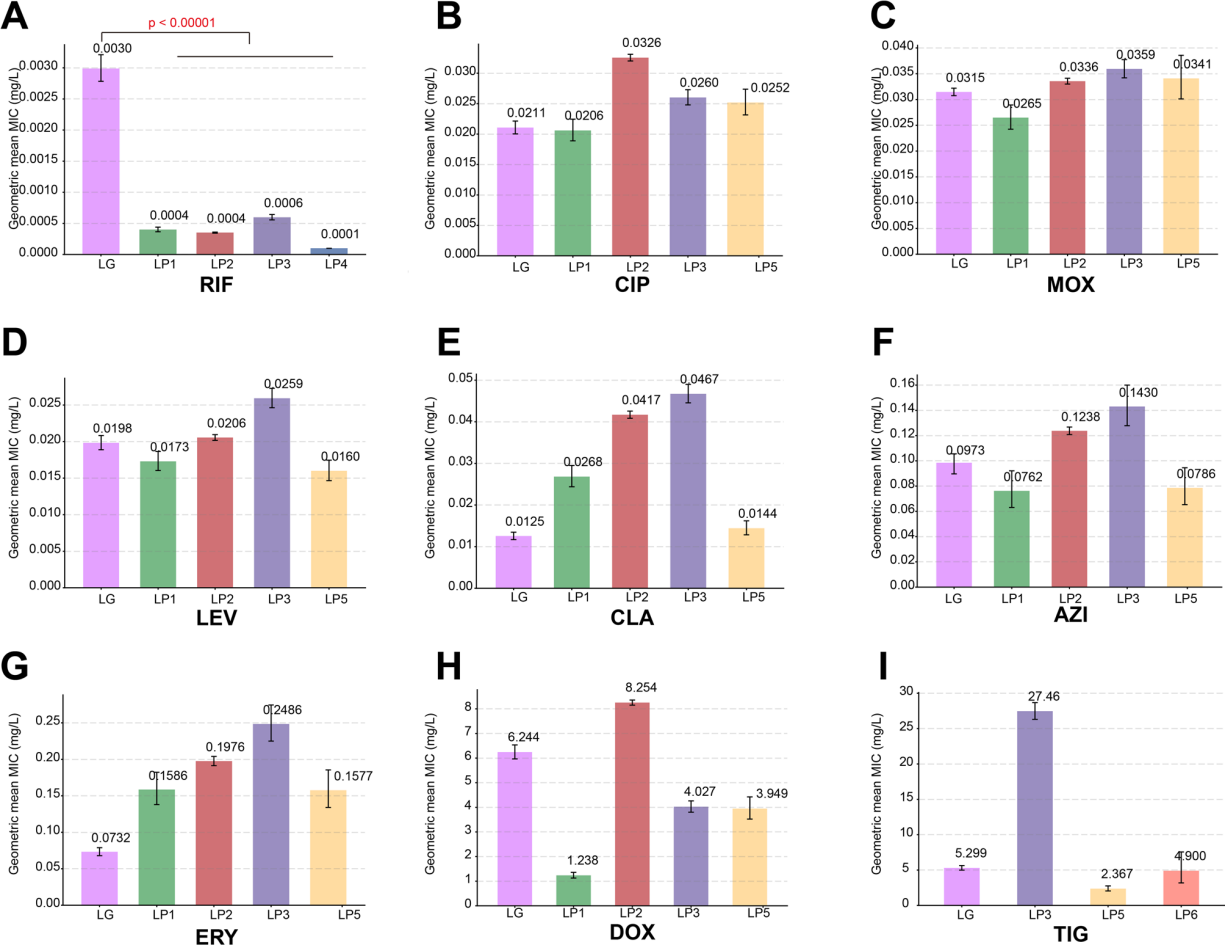
Comparison of geometric mean MICs for *L. gormanii* (LG) and *L. pneumophila* (LP). The bar charts display the geometric mean MICs in mg/L for nine antimicrobial agents. The purple bars (LG) represent the *L. gormanii* isolates from this study (*n* = 207), while the other bars (LP) correspond to various *L. pneumophila* datasets from published literature. Error bars indicate the 95% confidence interval (CI). Statistical significance was determined using a one-way ANOVA with Tukey’s HSD post hoc test on log-transformed MIC data. A significant difference was observed only for rifampin, where the geometric mean MIC for *L. gormanii* was substantially higher than all comparative *L. pneumophila* datasets (*p* < 0.00001). For the other eight antibiotics, the geometric mean MIC of *L. gormanii* was not higher than at least one *L. pneumophila* dataset; therefore, these statistical comparisons are not explicitly shown. LG: the present *L. gormanii* dataset; LP1: Vandewalle-Capo et al. dataset; LP2: Yang et al. dataset; LP3: Scaturro et al. dataset; LP4: Wilson et al. dataset; LP5: Minetti et al. dataset; LP6: Lang et al. dataset

The analysis revealed distinct susceptibility patterns between the two species (Table 2; Fig. 2). The most striking divergence was observed with rifampin. The geometric mean MIC for *L. gormanii* (0.0030 mg/L) was 5- to 30-fold higher than the reported means for *L. pneumophila* (0.0001–0.0006 mg/L), with the geometric mean showing a 9.58-fold higher when compared against the four published datasets (Fig. 2A). This suggests a lower intrinsic susceptibility in *L. gormanii.* In contrast, for most other tested agents, including the fluoroquinolones (CIP, MOX, and LEV), tetracyclines (DOX, and TIG), and the macrolides (AZI and CLA), the geometric mean MICs for *L. gormanii* were largely comparable to those of *L. pneumophila*, falling within established published ranges (Fig. 2). Notably, *L. gormanii* exhibited a slightly elevated susceptibility to ERY, with its geometric mean MIC (0.073 mg/L) being considerably lower than the values reported for *L. pneumophila* (Fig. 2G).

**Table 2:** Susceptibility of antimicrobials against *L.gormanii and L. pneumphila* by BMD method described by other articles

Antibiotics	Species	MIC_50_		MIC_90_	Geometrics mean MIC	MIC range	Number of isolates	Sources	Ref.
RIF	LP		0.0005	0.0005	0.0004	0.000063-0.002	1464	Env.	[21]
	LP		0.00045	0.0009	0.0006	0.0001-0.0035	204	Clin.	[22]
	LP		0.0005	0.0005	0.0004	0.00012-0.001	109	Clin.	[18]
	LP		0.0001	0.0001	0.0001	0.0001	92	Env.	[23]
	LG		0.004	0.004	0.0030	0.000125-0.008	207	Env.	This study
CIP	LP		0.031	0.063	0.0326	0.008-0.5	1464	Env.	[21]
	LP		0.03	0.03	0.0260	0.0075-0.06	204	Clin.	[22]
	LP		0.016	0.032	0.0206	0.008-0.064	109	Clin.	[18]
	LP		0.032	0.032	0.0252	0.016-0.125	107	Clin.	[24]
	LG		0.016	0.031	0.0211	0.0008-0.063	207	Env.	This study
MOX	LP		0.031	0.031	0.0336	0.016-0.25	1464	Env.	[21]
	LP		0.03	0.06	0.0359	0.03-0.5	204	Clin.	[22]
	LP		0.032	0.032	0.0265	0.008-0.064	109	Clin.	[18]
	LP		0.032	0.064	0.0341	0.008-0.25	107	Clin.	[24]
	LG		0.031	0.031	0.0315	0.016-0.063	207	Env.	This study
LEV	LP		0.016	0.031	0.0206	0.016-0.25	1464	Env.	[21]
	LP		0.03	0.03	0.0259	0.015-0.5	204	Clin.	[22]
	LP		0.016	0.032	0.0173	0.004-0.032	109	Clin.	[18]
	LP		0.06	0.032	0.0160	0.004-0.064	107	Clin.	[24]
	LG		0.016	0.031	0.0198	0.008-0.125	207	Env.	This study
CLA	LP		0.031	0.063	0.0417	0.004-0.125	1464	Env.	[21]
	LP		0.06	0.06	0.0467	0.015-0.06	204	Clin.	[22]
	LP		0.032	0.032	0.0268	0.004-0.064	109	Clin.	[18]
	LP		0.016	0.032	0.0144	0.002-0.064	107	Env.	[24]
	LG		0.016	0.016	0.0125	0.004-0.032	207	Env.	This study
AZI	LP		0.125	0.25	0.1238	0.008-1	1464	Env.	[21]
	LP		0.125	0.5	0.1430	0.03-1	204	Clin.	[22]
	LP		0.06	0.5	0.0762	0.015-2	109	Clin.	[18]
	LP		0.125	0.25	0.0786	0.008-2	107	Clin.	[24]
	LG		0.125	0.125	0.0973	0.004-0.5	207	Env.	This study
ERY	LP		0.25	0.5	0.1976	0.031-0.5	1464	Env.	[21]
	LP		0.5	0.5	0.2486	0.06-1	204	Clin.	[22]
	LP		0.125	0.5	0.1586	0.03-1	109	Clin.	[18]
	LP		0125	0.25	0.1577	0.032-1	107	Clin.	[24]
	LG		0.063	0.125	0.0732	0.016-0.25	207	Env.	This study
DOX	LP		8	8	8.254	2-16	1464	Env.	[21]
	LP		4	8	4.027	1-16	204	Clin.	[22]
	LP		1	2	1.238	0.12-2	109	Clin.	[18]
	LP		4	8	3.949	0.25-16	107	Clin.	[24]
	LG		8	8	6.244	4-8	207	Env.	This study
TIG*	LP		32	32	27.46	8-32	204	Clin.	[22]
	LP		2	8	2.367	0.25-8	107	Clin.+Env.	[24]
	LP		8	16	4.900	0.125-16	41	Clin.+Env.	[25]
	LG		4	8	5.299	0.25-16	207	Env.	This study
TMP/SMX#	LG		1	1	0.80	0.25-2	207	Env.	This study

*Env.* Environmental, *Clin.* Clinical, *LP* *L. pneumophila*, *LG* *L. gormanii*

* Scaturro et al., reported a relatively higher MIC (about 6-12-fold of geometrics mean) of TIG for *L. pneumophila* when compared with Minetti et al. and Lang et al., report. # A limited number of published studies to date (e.g., doi: 10.26444/aaem/167934 and doi: 10.1038/s41598-019-42425-1) have reported MIC data for TMP/SMX against *L. pneumophila* using the E-test method. Consequently, these studies were not included in the present comparison

## Discussion

The emergence of *L. gormanii* as a notable, albeit underdiagnosed, causative agent of legionellosis necessitates a thorough understanding of its antimicrobial susceptibility patterns to guide effective clinical management. This study provides the first comprehensive evaluation of the in vitro activity of ten representative antimicrobial agents against a substantial and geographically diverse collection of 207 environmental *L. gormanii* isolates from China. The principal findings reveal distinct susceptibility profiles, establish crucial ECOFFs, and highlight key differences compared to the more extensively studied *L. pneumophila*.

Our results confirm that rifampin exhibited the most potent in vitro activity against *L. gormanii*, with a geometric mean MIC of 0.0030 mg/L and an ECOFF of 0.016 mg/L. While this underscores its potential therapeutic utility, it is noteworthy that the geometric mean MIC values for rifampin against *L. gormanii* were approximately 5-30-fold higher than those typically reported for *L. pneumophila* [18, 21–23]. This observation suggests a potentially lower intrinsic susceptibility of *L. gormanii* to rifampin, which, while may still highly active in vivo, might warrants mechanistic investigation.

Among macrolides, clarithromycin was the most active agent against *L. gormanii* (geometric mean MIC = 0.0125 mg/L; ECOFF = 0.063 mg/L), displaying MIC values approximately three dilutions lower than azithromycin (geometric mean MIC = 0.0973 mg/L; ECOFF = 0.5 mg/L) and erythromycin (geometric mean MIC = 0.25 mg/L). This profile was similar with that observed in *L. pneumophila* (CLA has about 2-16-fold lower MICs compared with AZI and ERY). Current legionellosis treatment often favor azithromycin due to its pharmacokinetic profile, better medical outcomes for patients and efficacy against *L. pneumophila* [26, 27]. This may also happen in *L. gormanii* caused legionellosis. The overall susceptibility to macrolides observed in *L. gormanii* was comparable to, or potentially better than, that reported for *L. pneumophila*, reinforcing their role in empirical therapy regimens (Table 2).

Fluoroquinolones (CIP, LEV, MOX) demonstrated good and consistent in vitro activity. These results align with their established efficacy in treating legionellosis [10, 28–30], and support their continued use for infections caused by *L. gormanii*, especially those severe legionellosis, as fluoroquinolone-based antimicrobial regimens provide a superior mortality [31, 32]. The susceptibility patterns for fluoroquinolones in *L. gormanii* appear largely similar to those seen in *L. pneumophila*, suggesting reliable coverage by this class. Conversely, tetracyclines, particularly doxycycline (geometric mean MIC = 6.244 mg/L), exhibited the least in vitro activity among the tested agents. Tigecycline, while slightly more active than doxycycline in vitro, still presented a high geometric mean MIC of 5.299 mg/L. This low sensitivity is a critical finding, cautioning against the empirical use of tetracyclines, especially doxycycline, for suspected *L. gormanii* infections. The inability to establish an ECOFF for doxycycline due to limited data points at varying concentrations further underscores its poor performance against these isolates.

The establishment of species-specific ECOFFs for nine of the ten antimicrobials against *L. gormanii* represents a significant advancement. These ECOFFs (RIF 0.016 mg/L; CIP, LEV, CLA 0.063 mg/L; MOX 0.125 mg/L; ERY 0.25 mg/L; AZI 0.5 mg/L; TMP/SMX 4 mg/L; TIG 16 mg/L) provide essential tools for antimicrobial stewardship and surveillance, allowing for the differentiation of wild-type isolates from those potentially harboring acquired resistance mechanisms [11, 33]. This is crucial for monitoring resistance trends over time and informing public health interventions, especially considering the environmental resilience of *L. gormanii* and its potential exposure to sub-inhibitory concentrations of antimicrobials in aquatic ecosystems. The observed differences in susceptibility between *L. gormanii* and *L. pneumophila*, particularly for rifampin, may be attributable to inherent structural or mechanistic variations. These could include differences in drug target affinity (e.g., in the *rpoB* gene for rifampin) or the presence and activity of distinct efflux pump systems. Notably, the azithromycin susceptibility of *L. gormanii* closely mirrors that of lpeAB-negative *L. pneumophila* [21], strongly suggesting the absence of a homologous efflux pump in *L. gormanii.* Future genomic researches are warranted to investigate these hypotheses and clarify the molecular determinants of its antibiotic susceptibility.

A key limitation of this study is the environmental origin of our isolates. *L. gormanii*, like *L. pneumophila*, thrives in aquatic systems and is associated with biofilms and protozoan hosts [34]. These environments can expose bacteria to various antimicrobial stresses, including disinfectants and sub-inhibitory concentrations of antibiotics [35, 36]. The intrinsic resistance profiles observed in these environmental isolates may thus reflect adaptations necessary for survival in these complex ecological niches. While these environmental profiles are generally considered representative of the potential susceptibility of clinical isolates (as the environment is the source of infection), studies directly comparing the susceptibility of matched environmental and clinical *L. gormanii* isolates would be beneficial to confirm this assumption. Furthermore, our isolate collection, while large, was not sufficient for a robust statistical comparison of MIC distributions or to determine source-specific ECOFFs between different environmental sources (e.g., water vs. soil), which remains an area for future investigation.

In light of these findings, future research directions should include testing a larger collection of geographically diverse *L. gormanii* isolates, including clinical strains, to validate these ECOFFs and susceptibility patterns. Investigating the molecular mechanisms underlying the observed susceptibility difference between *L. gormanii* and *L. pneumophila* with rifampin, particularly the role of efflux pumps and potential target variations, is crucial, which may require intensive genomic research. Studies evaluating the intracellular activity of these agents against *L. gormanii* within host cells (e.g., amoebae or macrophages) would provide a more clinically relevant assessment of efficacy. While the ECOFFs for *L. gormanii* provide a valuable baseline, it is imperative to distinguish them from clinical breakpoints. These ECOFFs serve as an epidemiological tool to identify potential non-wild-type isolates and monitor for emerging resistance, rather than as a guide for clinical decision-making, as they do not account for PK/PD parameters or clinical efficacy data.

## Conclusions

This study establishes a crucial baseline for the antimicrobial susceptibility of *L. gormanii*, providing the first species-specific ECOFFs for nine key antimicrobials. These values are essential tools for surveillance programs monitoring for acquired resistance. Our findings highlight significant differences in susceptibility profiles compared to *L. pneumophila*, particularly for rifampin, suggesting that extrapolating treatment guidelines between these species may be inappropriate.

It must be emphasized that these findings are based on environmental isolates and the determination of ECOFFs; therefore, they are not directly translatable to clinical practice. The results should be considered preliminary, serving primarily to generate hypotheses and inform public health surveillance. They are not sufficient on their own to support immediate changes to clinical protocols. Future research, including comprehensive studies correlating in vitro data with genomic determinants, PK/PD parameters, and clinical outcomes, is essential to bridge the gap from epidemiological surveillance to the establishment of robust clinical breakpoints for *L. gormanii* infections.

## Supplementary Information

Below is the link to the electronic supplementary material.

Supplementary Material 1.

Supplementary Material 2.

## Data Availability

No datasets were generated or analysed during the current study.

## Abbreviations

MIC: Minimum inhibitory concentration
ECOFF: Epidemiological cutoff value
BMD: Broth microdilution
CIP: Ciprofloxacin
MOX: Moxifloxacin
LEV: Levofloxacin
ERY: Erythromycin
CLA: Clarithromycin
AZI: Azithromycin
TMP/SMX: Trimethoprim-sulfamethoxazole
DOX: Doxycycline
TIG: Tigecycline
RIF: Rifampin
BCYEα: Buffered charcoal yeast extract agar
BYE: Buffered yeast extract
AST: Antimicrobial susceptibility testing
EUCAST: European Committee on Antimicrobial Susceptibility Testing
PK/PD: Pharmacokinetic/pharmacodynamic
